# Antitumor effects of Chinese herbal medicine compounds and their nano-formulations on regulating the immune system microenvironment

**DOI:** 10.3389/fonc.2022.949332

**Published:** 2022-09-23

**Authors:** Kexiang Sun, Linguangjin Wu, Shuyun Wang, Wanli Deng

**Affiliations:** Department of Medical Oncology, Putuo Hospital, Shanghai University of Traditional Chinese Medicine, Shanghai, China

**Keywords:** oncology, traditional Chinese medicine, TCM, tumor immune microenvironment, nano-formulation

## Abstract

Traditional Chinese medicine (TCM), including herbal medicine, acupuncture and meditation, has a wide range of applications in China. In recent years, herbal compounding and active ingredients have been used to control tumor growth, reduce suffering, improve quality of life, and prolong the life span of cancer patients. To reduce side effects, herbal medicine can be used in conjunction with radiotherapy and chemotherapy or can be used as an adjuvant to strengthen the immune effect of anticancer vaccines. In particular, in the immunosuppressed tumor microenvironment, herbal medicine can have antitumor effects by stimulating the immune response. This paper reviews the advances in research on antitumor immunomodulation in Chinese herbal medicine, including the regulation of the innate immune system, which includes macrophages, MDSCs, and natural killer cells, and the adaptive immune system, which includes CD4+ T cells, CD8+ T cells, and regulatory T cells (Tregs), to influence tumor-associated inflammation. In addition, a combination of active ingredients of herbal medicine and modern nanotechnology alter the tumor immune microenvironment. In recent years, immunological antitumor therapy in TCM has been applied on a reasonably large scale both nationally and internationally, and there is potential for further clinical expansion. Investigation of immune modulation mechanisms in Chinese herbal medicine will provide novel perspectives of how herbal medicine controls tumor growth and metastasis, which will contribute to the evolution of tumor research.

**Methodology:**

Experimental research between the years of 2012-2022, meta-analysis and reviews for the period 2002-2022 found on the Databases including PubMed, Embase, and the Cochrane database were used. The inclusion criteria were experimental research literature addressing the anti-tumor immunological effects of active ingredients and nanoparticles in Chinese herbal medicine. Exclusion criteria were articles that addressed Chinese herbal medicine and nano-formulations without discussing anti-tumor immunological effects in innate, adaptive immune cells, MDSCs, and nuclear factors.

## Introduction

Worldwide, tumors have increased in their morbidity and associated mortality, constituting a grave risk to human health and life. According to contemporary research, the development of tumors is correlated with each individual’s immune system. Immunotherapy has been shown to be a powerful approach for treating a broad range of cancers and is widely used in clinical practice.

Given its large population, China accounts for approximately a quarter of the world’s new cancer cases and deaths, which leads to a serious burden of sickness (1). Traditional Chinese Medicine (TCM) is a unique diagnostic and therapeutic method that has been used for thousands of years and is widely used in China. Traditional herbal medicine is mostly used in the form of compound prescriptions in clinical oncology and includes oral herbal medicines, granules or capsules, and injections (2).

Numerous studies indicate that the combination of herbal medicine with antitumor therapy can achieve significant tumor suppression, reduce drug resistance, reduce adverse effects, and improve overall patient health and quality of life (3).

**Figure 1:**
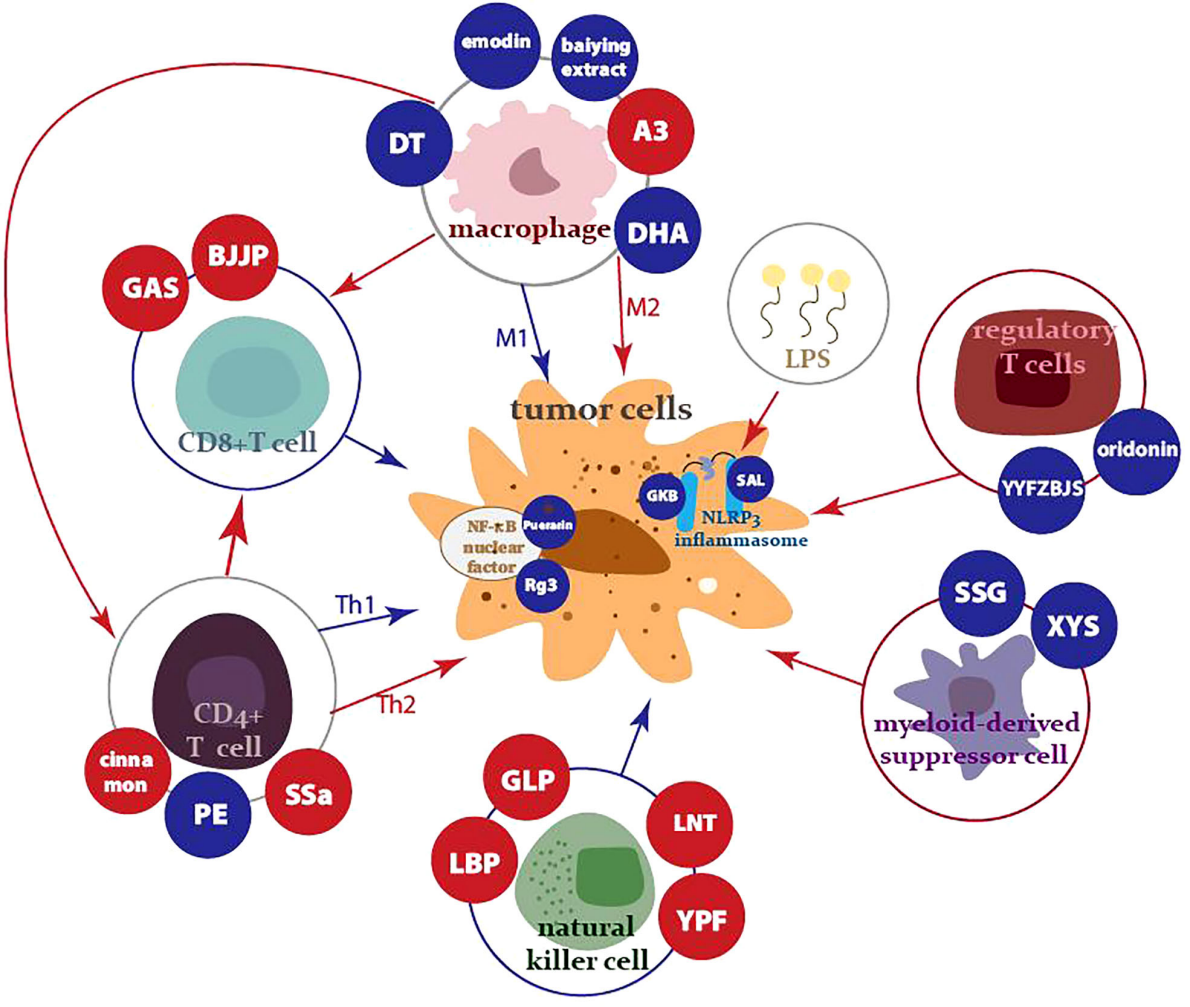
Innate, adaptive immune cells, MDSCs, and nuclear factors controlled by antitumor traditional herbal medicine and its components. The red arrows and dots represent activation, while the blue arrows and dots indicate suppression.

Various studies have demonstrated that herbal medicine can inhibit the proliferation ^1^ and metastasis of a variety of tumor cells, including lung (4), breast (5), colorectal (6), and other tumor cells, while facilitating their apoptosis. This paper will review the immunomodulatory role of herbal medicine in tumor immunity, including in regulation of the innate immune system, the adaptive immune system, and tumor-associated inflammation, as well as the emerging strengths of integrating herbal medicine with modern nanotechnology (**Figure 1**).

## Regulation of the innate immune system

### Macrophages

Substantial clinical and experimental evidence indicates that macrophages, which are present abundantly in most tumor types, play a major regulatory role in promoting tumor progression to malignancy (7). The presence of tumor-associated macrophages (TAMs) is generally associated with a poor prognosis in solid tumors.

There are two main categories of activated macrophages: classically activated macrophages (M1; interferon (IFN)-γ/lipopolysaccharide (LPS)-dependent) and alternatively activated macrophages (M2; interleukin (IL)-4)/IL-13/IL-10-dependent) (8).

M1 macrophages have a high capacity for antigen presentation and the ability to secrete a range of cytokines and chemokines that promote inflammatory responses against pathogens and tumor cells as well as mediate the killing of tumor cells. In comparison, M2 macrophages have low antigen-presenting activity and have the functions of suppressing inflammatory responses and enhancing wound healing, angiogenesis, and tissue repair (9).

M1 macrophages support oncogenesis by inducing oxidative stress and an anti-apoptotic response, leading to cell survival and proliferation. During the chronic inflammatory process, M1 macrophages influence the tumor microenvironment and sustain further tumorigenesis. M2 cells also drive tumorigenesis by regulating the formation of extracellular matrix and angiogenesis in the tumor mesenchyme. In contrast, there is some evidence of tumor-suppressive actions of macrophages (10). Yuan R et al. (11) found that an M2 phenotype predominates in TAMs and plays a critical role in promoting tumor growth, invasion, and metastasis.

Evidence indicates that in nascent tumors, TAMs display an M1-like phenotype and can eliminate some immunogenic tumor cells. Subsequently, tumor progression is associated with skewing and subversion of macrophage function by the cues within the tumor microenvironment that could direct an M2-like polarization of TAMs that is pro-tumorigenic (12).

Typically, suppression of M2 polarization may help to stimulate antitumor-specific immune responses and decrease tumor metastasis. Many works have shown that herbal medicine could control the growth and metastasis of tumors by moderating M1/M2 macrophages.

Epithelial-mesenchymal transition (EMT) contributes to cancer progression, particularly metastasis. The reprogramming of gene expression during EMT is triggered and controlled by multiple signaling pathways, in which transforming growth factor-β (TGF-β) signaling plays a primary role (13). M2 macrophages secretes TGF-β1, leading to proliferation, invasion, and migration of tumor cells. Emodin is an anthraquinone compound isolated from Chinese herbs such as *Rheum palmatum* L., *Rheum tanguticum* Maxim. ex Bal£. and *Rheum officinale* Bail1. (The effects and specific information for each herbal medicine are shown in **Tables 2**, **3**).

**Table 1:** Abbreviations and terminology.

**TCM**	Traditional Chinese Medicine	**MDSC**	myeloid-derived suppressor cell
**Treg**	regulatory T cell	**M1**	classically activated macrophages
**M2**	alternatively activated macrophages	**TAM**	tumor-associated macrophage
**EMT**	epithelial-mesenchymal transition	**TGF**	transforming growth factor
**CSC**	cancer stem cell	**TIC**	tumor-initiating cell
**SLT**	*Solanum lyratum Thunb*	**A3**	anemoside A3
**TNF**	tumor necrosis factor	**IL**	interleukin
**TLR**	toll-like receptor	**MAPK**	mitogen-activated protein kinase
**VEGF**	vascular endothelial growth factor	**NHSCC**	neck and head squamous cell carcinoma
**DHA**	dihydroartemisinin	**STAT**	signal transducer and activator of transcription
**DT**	dihydroisotanshinone I	**CCL**	C-C motif ligand
**CXCL**	C-X-C motif chemokine ligand	**G-MDSC**	granulocyte-like MDSC
**M-MDSC**	monocyte-like MDSC	**HCC**	hepatocellular carcinoma
**MiRNA**	microRNA	**CtBP**	carboxy terminal binding protein
**SSG**	Shuangshen granules	**XYS**	Xiaoyaosan
**MMP**	matrix metalloproteinase	**NK**	natural killer
**GFP**	*G. frondosa* polysaccharide	**LNT**	lentinan
**GLP**	*G. lucidum* polysaccharide	**LBP**	*Lycium barbarum* polysaccharide
**YG**	yeast glucan	**SMG**	simulated microgravity conditions
**IFN**	interferon	**NO**	nitrogen mo*no*xide
**YPF**	Yupingfeng	**NSCLC**	non-small cell lung cancer
**Th**	T-helper	**SSa**	Saikosaponin A
**BJJP**	Biejiajian pill	**GAS**	gastrodin
**APC**	antigen-presenting cell	**CTLA4**	cytotoxic T-lymphocyte-associated protein 4
**PAMP**	pathogen-associated molecular pattern	**DAMP**	damage-associated molecular patterns
**LPS**	lipopolysaccharide	**SAL**	salidroside
**AMPK**	adenosine 5’-monophosphate (AMP)-activated protein kinase	**ROS**	reactive oxygen species
**GKB**	Ginkgolide B	**LC3B**	microtubule-associated protein 1 light chain 3 beta
**PCNA**	proliferating cell nuclear antigen	**NF-kB**	nuclear factor kappa B
**Rg3**	compound 20 (R)-ginsenoside	**IKK**	inhibitor of kappa B kinase
**CSO**	coix seed oil	**NG**	Naringin
**NLC**	nanostructured lipid carrier	**NDNLC**	neodecanoic triglyceride nanostructured lipid carrier
**NONLC**	oleic acid nanostructured lipid carrier	**NCNLC**	NG + CSO nanostructured lipid carrier
**DOX**	doxorubicin	**pCa**	prostate cancer
**PSMA**	prostate-specific membrane antigen	**TAN**	Tanshinone
**ATO**	As2O3	**RGDyC**	RGDArg-Gly-Asp
**PAMAM**	polyamidoamine	**NPs**	metal and/or metal oxide nanoparticles
**ADR**	adverse drug reactions	**AAS**	aristolochic acid
**AAN**	aristolochic acid nephropathy	**DC**	dendritic cell
**TCCM**	tumor cell-conditioned medium	**TNBC**	triple-negative breast cancer

**Table 2:** Summary of the herbal active ingredients’ immunomodulating effects referred to in this review.

	Chinese Medicine Compound	Immune efficacy	Cancer type	*In vivo* or *in vitro*	Ref.
**Macrophages**	Emodin	↓M2, TGF-β1, CD44^+^/CD24^-^ CSCs ↓FoxC2, Nanog, Oct4 and KLF4 ↑CD4^+^ and CD8^+^ T cells	Breast cancer	*In vivo*, *in vitro*	(14)
	Baiying extract	↑M0→M1, ↓M0→M2 ↓CDK4, Cyclin D1, p21, E-cadherin, cleaved caspase-3, and cleaved PARP ↓CD206^+^ ↑CD86^+^ cells	Ovarian cancer	*In vitro*	(15)
	Anemoside A3	↑M0→M1 ↑CD86^+^ cells ↑TNF-α, and IL-12	Breast cancer	*In vivo*, *in vitro*	(16)
	Dihydroartemisinin	↓M2 ↓IL-10, VEGFA, MMP-9, and MMP-10	Neck squamous cell carcinoma	*In vivo*, *in vitro*	(17)
	Dihydroisotanshinone I	↓CCL2,CXCL1 ↑IL-8	Lung cancer	*In vivo*, *in vitro*	(18)
**MDSCs **	Shuangshen granules	↓MDSCs ↓mTOR, S6K1, and Myc ↓GFP^+^ LLC cells	Lung cancer	*In vivo*, *in vitro*	(19)
	Xiaoyaosan	↓CD11b^+^F4/80^+^ macrophage, CD11b^+^Gr^lo^Ly6C^hi^ cells ↓TGF-β, IL-6, MMP-9,VEGF mRNA, CD31	Colorectal cancer	*In vivo*	(20)
**Natural killer (NK) cells**	G. lucidum polysaccharide Lycium barbarum polysaccharide	↑NK cells, IFN-γ, perforin and granzyme-B ↑NKp30, ↑receptor NKG2D	Myelogenous leukemia	*In vitro*	(21)
	Lentinan	↑NK cells ↑NKp30			
	Yupingfeng	↑NK cells ↑IL-12 ↓TGF-β, IDO and IL-10.	Neck squamous cell carcinoma	*In vivo*, *in vitro*	(22)
**CD4^+^ T cells**	Saikosaponin A	↑IFN-γ, IL-12, STAT4 ↓IL-4, IL-10	Breast Cancer	*In vivo*, *in vitro*	(23)
	Cinnamon	↑Th1, Tc1, T-bet ↓Th17, Treg, Foxp3	Melanoma	*In vivo*	(24)
	*Pinellia ternata* lipid-soluble extract	↑CD4^+^Tcells ↓Th2 ↑beta-catenin and c-myc	Cervical cancer	*In vivo, in vitro*	(25)
**CD8^+^ T cells**	Biejiajian pill	↑CD8^+^ T cells ↑TNF-α and IFN-γ ↑CCL5	Hepatocellular carcinoma	*In vivo*, *in vitro*	(26)
	Gastrodin	↑CD8^+^ T cells ↑CD80, CD86 and MHCI, APCs ↑IL-12 and TNF-α	Melanoma	*In vivo*, *in vitro*	(27)
**Regulatory T cells** **(Tregs)**	Oridonin	↓Tregs ↑CD8^+^ T cells ↓CTLA4, CD69, phosphorylation of Smad2 and Smad3,TGF-βRI and TGF-βRII ↑TGF-β1 and IL-10	Breast cancer	*In vivo*, *in vitro*	(28)
	Yi-yi-fu-zi-bai-jiang-san	↓Tregs ↓Ki67, PCNA, and BrdU ↑IL-17A and TNF-α ↓Foxp3-mRNA, β-catenin	Colorectal cancer	*In vivo*, *in vitro*	(29)
**NLRP3** **Inflammasome**	Salidroside	↑AMPK phosphorylation ↓NLRP3, caspase-1, pro-caspase-1,pro-IL-1β, and IL-1β	Lung cancer	*In vitro*	(30)
	Ginkgolide B (GKB)	↓NLRP3 ↑beclin-1, p62, and LC3B ↓PCNA and Bcl-2	Lung cancer	*In vitro*	(31)
**NF-κB**	Puerarin	↓CCR7, CXCR4, MMP-2 and MMP-9 ↓collagen I, collagen IV and fibronectin ↓VCAM-1 and ICAM-1 ↓NF-κB pathway	Breast cancer	*In vitro*	(32)
	Compound 20 (R)-ginsenoside (Rg3)	↓p-p65, p65, p-IKK, and IKK ↑caspase-3, -8, -9 and Bax ↓Bcl-2 and survivin ↓NF-κB pathway	Neck squamous cell carcinoma	*In vitro*	(33)

*A3, Anemoside A3, DHA,Dihydroartemisinin, DT, Dihydroisotanshinone I, SSG, Shuangshen granules, XYS, Xiaoyaosan, GLP ,G. lucidum polysaccharide, LBP, Lycium barbarum polysaccharide, LNT, lentinan, YPF, Yupingfeng, SSa, Saikosaponin A, PE, Pinellia ternata lipid-soluble extractBJJP, Biejiajian pill, GAS, Gastrodin, SAL, Salidroside, YYFZBJS, Yi-Yi-Fu-Zi-Bai-Jiang-San, GKB, Ginkgolide B, Rg3, Compound 20 (R)-ginsenosid.

↑ implies an increase in the relevant level and ↓ implies a decrease in the relevant level.

**Table 3:** List of Chinese herbs, Latin binomials, active ingredients, and species mentioned in the review.

Chinese name			Latin binomial	Active ingredients	Family
**Dahuang**			*Rheum palmatum* L. *Rheum tanguticum* Maxim. ex Bal£. *Rheum officinale* Bail1.	Emodin	Polygonaceae
**Baiying**			*Solanum lyratum* Thunb.		Solanaceae
**Baitouweng**			*Pulsatilla chinensis* (Bge.)Regel	Anemoside A3	Ranunculaceae
**Qinghao**			*Artemisia annua* L.	Dihydroartemisinin	Compositae
**Danshen**			*Salvia miltiorrhiza* Bge.	Dihydroisotanshinone I	Lamiaceae
**Shuangshen** **granules** **(SSGs)**	Xiyangshen		*Panax quinquefolium* L.	American ginseng polysaccharide, American ginseng ginsenoside	Araliaceae
	Sanqi		*Panax notoginseng* (Burk) F. H. Chen	Panax notoginseng saponins	Araliaceae
	Dongchongxiacao		*Rabdosia rubescens* (Hemsl.)Hara	Cordycepin	Ergotaceae
**Xiaoyaosan** **(XYS)**	Chaihu		*Bupleurum chinense DC.* *Bupleurum scorzonerifolium* Willd.	Saikosaponin A	Umbelliferae
	Danggui		*Angelica sinensis* (Oliv.) Diels.	A. sinensis polysaccharides	Umbelliferae
	Baishao		*Paeonia lactiflora* Pall.	Paeoniflorin	Paeoniaceae
	Baizhu		*Atractylodes macrocephala* Koidz.	Atractylenolide	Compositae
	Fuling		*Poria cocos* (Schw.)Wolf	Poria cocos triterpenes Poria cocos polysaccharides	Polyporaceae
	Gancao		*Glycyrrhiza uralensis* Fisch. *Glycyrrhiza inflata* Bat. *Glycyrrhiza glabra* L.	Licorice polysaccharides	Leguminosae
	Bohe		*Mentha haplocalyx* Briq.	Menthol	Lamiaceae
	Shengjiang		*Zingiber officinale* Rosc.	Gingerols, shogaols	Zingiberaceae
**Lingzhi**			*Ganoderma lucidum* (Leyss.ex Fr.)Karst. *Ganoderma sinense* Zhao, Xu et Zhang	G. lucidum polysaccharide	Polyporaceae
**Gouqi**			*Lycium barbarum* L.	Lycium barbarum polysaccharide	Solanaceae
**Yupingfeng** **(YPF)**	Huangqi		*Astragalus membranaceus* (Fisch.)Bge.	Astragaloside, Astragalus polysaccharides	Papilionaceae
	Baizhu		*Atractylodes macrocephala* Koidz.	Atractylenolide	Compositae
	Fangfeng		*Saposhnikovia divaricata* (Turcz.) Schischk.	Polysaccharides	Umbelliferae
**Rougui**			*Cinnamomum cassia* Presl	Cinnamic aldehyde	Lauraceae
**Banxia**			*Pinellia ternata* (Thunb.) Breit.	Amino acids, volatile oils, alkaloids, pinellia protein, organic acids, steroids	Araceae
**Chaihu**			*Bupleurum chinense* DC. *Bupleurum scorzonerifolium* Willd.	Saikosaponin A	Umbelliferae
**Biejiajian Pill**	Biejiajiao		*Trionyx sinensis* Wiegmann		Turtle family
	Ejiao		*Equus asinus* L.		Equidae
	Fengfang		*Saposhnikovia divaricata* (Turcz.) Schischk.	Polysaccharides	Umbelliferae
	Shufuchong		*Porcellio scaber* Latreille		Armadillididae
	Tubiechong		*Eupolyphaga sinensis* Walker *Steleophaga plancyi* (Boleny)		Periplaneta
	Qianglang		*Catharsius molossus* L.		Scarabidae
	Xiaoshi			Na_2_SO_4_·10H_2_O	Glauber’s salt
	Chaihu		*Bupleurum chinense* DC. *Bupleurum scorzonerifolium* Willd.	Saikosaponin A	Umbelliferae
	Huangqin		*Scutellaria baicalensis* Georgi	Baicalein	Lamiaceae
	Banxia		*Pinellia ternata* (Thunb.) Breit.	Amino acids, volatile oils, alkaloids, pinellia protein, organic acids, steroids	Araceae
	Dangshen		*Codonopsis pilosula* (Franch.)Nannf. *Codonopsis pilosula* Nannf. var. modesta (Nannf.) L.T.Shen *Codonopsis tangshen* Oliv.	*Codonopsis pilosula* polysaccharides	Campanulaceae
	Ganjiang		*Zingiber officinale* Rosc.	Gingerol	Zingiberaceae
	Houpu		*Magnolia officinalis* Rehd.et Wils. *Magnolia officinalis* Rehd.et Wils.var.biloba Rehd.et Wils.	Magnolol, honokiol	Magnoliaceae
	Guizhi		*Cinnamomum cassia* Presl.	Cinnamaldehyde	Lauraceae
	Baishao		*Paeonia lactiflora* Pall.	*Paeoniae radix alba* polysaccharides	Ranunculaceae
	Shegan		*Belamcanda chinensis* (L.) DC.	Flavonoids, volatile oils, phenolic acids, steroids, triterpenoids	Iridaceae
	Taoren		*Prunus persica* (L.)Batsch *Prunus davidiana* (Carr.) Franch.	Amygdalin	Rosaceae
	Mudanpi		*Paeonia suffruticosa* Andr.	Moutan Cortex polysaccharides	Ranunculaceae
	Dahuang		*Rheum palmatum* L. *Rheum tanguticum* Maxim. ex Bal£. *Rheum officinale* Bail1.	Emodin	Polygonaceae
	Lingxiaohua		*Campsis grandiflora* (Thunb.) K. Schum. *Campsis radicans* (L.)Seem.	Fatty alcohols, fatty ketones, fatty acids, phenolic acids, cyclohexylethanoids	Chaveidae
	Tinglizi		*Descurainia sophia* (L.) Webb. ex Prantl. *Lepidium apetalum* Willd.	Glucocappasalin	Bignoniaceae
	Shiwei		*Pyrrosia sheareri* (Bak.) Ching *Pyrrosia lingua* (Thunb.) Farwell *Pyrrosia petiolosa* (Christ) Ching		Polypodiaceae
	Qumai		*Dianthus superbus* L. *Dianthus chinensis* L.		Caryophyllaceae
**Tianma**			*Gastrodia elata* Bl.	Gastrodin	Orchid
**Donglingcao**			*Rabdosia rubescens* (Hemsl.) Hara	Oridonin	Lamiaceae
**Yi-yi-fu-zi-bai-jiang-san**		**Yiyiren**	*Coix lacryma-jobi* L. var.ma-yuen (Roman.) Stapf	Coix seed polysaccharides	Gramineae
		**Fuzi**	*Aconitum carmichaelii* Debx.	Aconitine	Ranunculaceae
		**Baijiangcao**	*Patrinia scabiosaefolia* Fisch. Ex Link., *P. villosa* (Thunb.) Juss.	Flavonoids, triterpenoids, organic acids	Valerianaceae
**Hongjingtian**			*Rhodiola crenulata* (Hook. f. et Thoms.) H. Ohba	Salidroside	Crassulaceae
**Yinxingye**			*Ginkgo biloba* L.	Ginkgolide B	Ginkgoaceae
**Gegen**			*Pueraria lobata* (Willd.)Ohwi.	Puerarin	Legumes
**Renshen**			*Panax ginseng* C. A. Mey.	Compound 20 (R)- ginsenoside	Araliaceae
**Yiyiren**			*Coix lacryma-jobi* L. var.ma-yuen (Roman.) Stapf	Coix seed polysaccharides	Gramineae
**Pishuang**				As2O3	Inorganic Arsenic
**Madouling**			*Aristolochia debilis* Sieb. et Zucc	Aristolochic acids	Aristolochiaceae
**Wutou**			*Aconitum carmichaeli* Debeaux	Aconitine	Ranunculaceae

*A3, anemoside A3; DHA, dihydroartemisinin; DT, dihydroisotanshinone I; GLP, G. lucidum polysaccharide; LBP, Lycium barbarum polysaccharide; GCP, glucocappasalin; GAS, gastrodin; SAL, salidroside; GKB, Ginkgolide B; YYFZBJS, Yi-yi-fu-zi-bai-jiang-san; Rg3, compound 20 (R)-ginsenoside; ATO, As2O3; AASs, aristolochic acid.

To examine the relationship between macrophage abundance and other properties in breast tumors, Liu Q et al. (14) examined data from 1,105 samples obtained from 1,098 patients with breast cancer from The Cancer Genome Atlas of breast tumors, and 529 samples were selected from these.

Using the expression level of the pan-macrophage marker CD68 as an indicator of macrophage abundance, they sorted CD68^lo^ and CD68^hi^ samples and found that the CD68^hi^ group was associated with pro-tumorigenic markers, including TGF-β1, EMT markers, stemness markers, and related transcription factors. These findings were confirmed by subsequent statistical analysis.

Subsequently, they found that emodin was able to reduce TGF-β1 expression and reverse the increased N-cadherin and reduced E-cadherin protein levels caused by TGF-β1 treatment. They isolated primary macrophages from mice, pretreated them with tumor cell-conditioned medium (TCCM) to generate TAM-like macrophages, and then added them to breast cancer cells. TCCM-pretreated macrophages exhibited production of TGF-β1 and an increased expression of CD206, a marker of M2 macrophages, which was attenuated by emodin. In addition, emodin significantly reduced both baseline peritoneal macrophage-conditioned medium (PMCM) treatment-induced TGF-β1 production in cancer cells and TGF-β1 induced Arg-1 expression, which has been shown to define immunosuppressive subsets of TAMs.

Thus, emodin was found to suppress cancer cell-induced macrophage M2-like polarization, inhibit polarized macrophage-induced TGF-β1 production in breast cancer cells, and block EMT of breast cancer cells induced by TGF-β1 from both macrophages and cancer cells.

As demonstrated by a wound-healing migration assay and Matrigel invasion assay, rhodopsin markedly reduced the migration and invasion of TGF-β1-enhanced breast cancer cells.

Self-renewing cancer stem cells (CSCs) and progenitor cells constitute a minor portion of neoplastic cells within tumors and are collectively defined as tumor-initiating cells (TICs). The TIC population is the key source of metastatic lesions in breast cancer.

Emodin reduced baseline levels of CD44^hi/+^/CD24^lo/-^, which are recognized CSC markers, and blocked TGF-β1-induced CSC increasing in 4T1 cells. The TME provides WNT and EGF signals contributed to tumor regeneration and therapy resistance. For primary PyMT tumor cells, emodin suppressed the percentage of progenitor cells and the average CD61 expression levels, a marker of progenitor population in breast cancer. The baseline expression of FoxC2, Nanog, Oct4 and KLF4 was downregulated by emodin in MDA-MB-231 cells, and the TGF-β1-induced expression of Jagged1, KLF4 and FoxC2 was decreased by emodin in 4T1 cells, which are all TIC-related.

Breast cancer TICs can be propagated *in vitro* as nonadherent spheres; these spherical clusters of self-replicating cells formed in suspension cultures are called mammospheres. Emodin significantly reduced both primary and secondary mammosphere formation in EO771, 4T1, MCF7, and MDA-MB-231 cells. Similarly, C57BL/6 mice transplanted with a prepared emodin EO771 single-cell showed a reduced estimated stem cell frequency, and MDA-MB-231 cells in NOD SCID mice remained effective. Both the *in vitro* mammosphere formation assays and the *in vivo* limiting dilution assays demonstrated that emodin reduced breast cancer TICs.

After BALB/C mice were inoculated with 4T1 cells, treatment with emodin notably reduced CD206^+^ M2-like macrophage infiltration and average cell surface CD206 level of macrophages. In addition, emodin treatment increased both CD4^+^ and CD8^+^ T cells in the tumors and the percentage of IFNγ^+^ cells. Emodin treatment decreased CD44^+^/CD24^-^ CSCs in the tumors and reduced the expression of EMT markers vimentin and MMP9.

EMT and TICs are exploited as therapeutic targets for metastatic breast cancer. Circulating tumor cells (CTCs) are pioneering tumor cells with stemness properties transendothelially and acquire mesenchymal characteristics following EMT, migrating into the intratumoral microvessels. Thereafter, CTCs can form new tumors and are a sign of breast cancer metastasis. *In vivo* experiments have confirmed that emodin substantially reduced lung metastasis and increased survival compared with the vehicle-treated control.

Emodin significantly inhibited Smad2/3 phosphorylation and nuclear translocation and suppressed TGF-β1-induced phosphorylation of AKT and the induction of Zeb1 and Twist by TGF-1β by TGF-β1 in both 4T1 cells and MDA-MB-231 cells, suggesting that emodin blocks TGF-β1-mediated crosstalk between TAMs and breast cancer cells.

Furthermore, Pourhajibagher M et al. (34) synthesized a transfersome form of nano-emodin as a novel sono-responsive nanomaterial that could significantly increase expression of caspase-3 and caspase-9, promoting cell apoptosis for head and neck squamous cell carcinoma. Another aloe emodin-encapsulated nanoliposome (35) was used to transfect the r-caspase-3 gene, leading to inhibition of the proliferation rate and an increased apoptotic rate in human gastric cancer cells.

In TCM, Baiying is used to treat malaria, jaundice, edema, gonorrhea, rheumatic joint pain, dampness, and furuncles. Baiying (*Solanum lyratum* Thunb.) extract (15) (SLT) significantly inhibited the cell viabilities and reduced the protein levels of CDK4 and cyclin D1 in both A2780 and SKOV3 cells. SLT inhibited cell proliferation and EMT and induced cell apoptosis, manifested as increased expression of p21, E-cadherin, cleaved caspase-3, and cleaved PARP and decreased expression of N-cadherin. In addition, SLT can activate ROS/p53 to regulate ovarian cancer progression.

The percentage of CD206^+^ M2 macrophages was significantly decreased in the A2780/SKOV3+M0 macrophage groups treated with SLT, while the percentage of CD86^+^ M1 macrophages was significantly increased, indicating that SLT could activate the polarization of M0 macrophages to M1 macrophages but inhibit the polarization of M0 macrophages to M2 macrophages, thus regulating the tumor immune microenvironment and inhibiting ovarian cancer progression.

Anemoside A3 (16) (A3) is an active compound from *Pulsatilla saponins* (Bge.) Regel, which can active the TLR4/NF-κB/MAPK signaling pathway. *In vitro* A3 increased the expression of CD86^+^ M0 macrophages, polarizing M0 macrophages into the M1phenotype and elevated TNF-α and IL-12 expression in a TLR4-dependent manner. Moreover, in a 4T1 murine breast cancer model, A3 induced M1 macrophage polarization and effectively inhibited tumor growth and tumor angiogenesis *in vivo*.

M2 phagocytosis promotes the progression of neck and head squamous cell carcinoma with tumor cell invasion, migration, and angiogenesis. Dihydroartemisinin (DHA) is a semi-synthetic derivative of *Artemisia annua* L., which has the function of clearing heat and relieving malaria and itching. In *in vitro* experiments, DHA (17) led to a significant decrease in the number of M2-like TAMs and reduced IL-10, vascular endothelial growth factor A (VEGFA), MMP-9, and MMP-10 expression. In addition, DHA decreased M2-like TAM-promoted vascular formation by inhibiting phosphorylation of STAT3.

The Chinese herb danshen (*Salvia miltiorrhiza* Bge.) is used to treat chest pain, abdominal pain, insomnia, and menstrual disorders. Clinical data analysis has revealed that treatment with salvia is highly correlated with decreased mortality in patients. Dihydroisotanshinone I (DT) is a fat-soluble phenanthrene compound extracted from the Chinese herb *Salvia miltiorrhiza* Bge. DT (18) treatment resulted in decreased expression of CCL2 and C-X-C motif chemokine ligand 1 (CXCL1) and increased expression of IL-8, blocking the ability of macrophages to promote lung cancer cell migration and the recruitment of macrophages by lung cancer cells. Furthermore, DT treatment significantly inhibited the final tumor volume in a xenograft nude mouse mode *in vivo* experiment.

Han S et al. (36) used a poly (D,L-lactic-co-glycolic acid) (PLGA)-based nanoparticle (NP) to co-encapsulate dihydrotanshinone I (DIH) and two other drugs, generating chemo-immunotherapeutic effects to reverse an immunosuppressive tumor microenvironment and found significantly increased survival among mice with hepatocellular carcinoma (HCC), without associated toxic signs.

### MDSCs

Myeloid-derived suppressor cells (MDSCs) are a population of cells of bone marrow origin with strong immunosuppressive properties that are present in the peripheral blood and tumor tissue of tumor-bearing mice or patients. In normal conditions, this population of cells differentiates into dendritic cells, macrophages, and granulocytes, but in the presence of inflammation, tumors, trauma, and infection, MDSCs can be significantly expanded.

MDSCs can be classified into two subgroups: granulocyte-like MDSCs (G-MDSCs) and monocyte-like MDSCs (M-MDSCs). In most experimental tumor animal models, G-MDSCs have a greater tendency to proliferate than M-MDSCs; hence, G-MDSCs are the predominant type of MDSCs in tumor tissue (37). In clinical practice, the accumulation of MDSCs in the peripheral blood and tumors of patients with liver cancer is strongly associated with HCC staging and poor survival, and the recruitment of MDSCs can cause liver tumor recurrence after transplantation (38). A high level of MDSCs in patients is often a sign of ineffective immunotherapy, and herbal medicine is often used for its antitumor effects to reduce the recruitment of MDSCs.

Immunosuppression is a dominant feature of MDSCs. Although MDSCs are implicated in the suppression of different cells of the immune system, the main target of MDSCs is T cells. MDSCs also impact the tumor microenvironment reformulation and tumor angiogenesis through VEGF expression, thus contributing to tumor development (39). In parallel, MDSCs can trigger the expression of miRNA101 in cancer cells. MiRNA101 subsequently represses CtBP2, which directly targets core stem cell genes, leading to increased stalkiness of cancer cells as well as higher metastatic and tumorigenic potential (40).

Shuangshen granules (SSGs) are composed of *Panax quinquefolium* L., *Panax notoginseng* (Burk) F. H. Chen and *Rabdosia rubescens* (Hemsl.) Hara. In a co-culture model *in vitro*, SSG treatment (19) exhibited a significant attenuating effect on bone marrow cell differentiation into CD11b^+^Ly6C^+^Ly6G^+^ cells and a partial attenuating effect on differentiation into CD11b^+^Ly6C^+^Ly6G^–^ cells in a dose-dependent manner. SSGs suppressed the mTOR, S6K1, and Myc protein levels both *in vivo* and *in vitro.* Next, the effect of SSGs on tumor growth and lung metastasis was assessed *in vivo*, and mice treated with SSGs showed a reduction in tumor weight and GFP^+^ LLC cells in lung tissue. The results showed that SSGs can attenuate the differentiation of bone marrow by inhibiting the mTOR signal pathway in the bone marrow microenvironment and decrease the systemic levels of MDSCs in the bone marrow, blood, and lungs, suggesting that SSGs not only have an inhibitory effect on tumor cell proliferation but also have a protective effect against lung metastasis.

Chronic restraint stress is one of the most recognized Liver Qi stagnation models as it may mimic symptoms of depression in humans and can promote angiogenesis, resulting in cancer development and impacting the migration of tumor cells (41). Xiaoyaosan (XYS) is a traditional Chinese medical formula consisting of Chaihu (*Bupleurum chinense* DC., *Bupleurum scorzonerifolium* Willd.), Danggui (*Angelica sinensis* (Oliv.) Diels.), Shaoyao (*Paeonia lactiflora* Pall.), Baizhu (*Atractylodes macrocephala* Koidz.), Fuling (*Poria cocos*(Schw.)Wolf.), Gancao (*Glycyrrhiza uralensis* Fisch., *Glycyrrhiza inflata* Bat., Glycyrrhiza glabra L.), Bohe (*Mentha haplocalyx* Briq.), and Shengjiang (*Zingiber officinale* Rosc.), which has been studied for its effectiveness in treating hepatitis (42).

Zhao L et al. (20) found that in an *in vivo* experiment, XYS-treated mice had fewer liver metastases and a lower liver weight, and XYS could significantly decrease the CD11b^+^F4/80^+^ macrophage population in the primary tumor tissue, accompanied by a significant reduction in CD11b^+^Gr^lo^Ly6C^hi^ cells. Treatment of mice undergoing chronic restraint stress with XYS significantly reduced TGF-β, IL-6, MMP-9, and VEGF mRNA and CD31 protein expression compared with mice in the blank-stress (BS) group, which indicated that XYS treatment reduced the effect of chronic restraint stress on the facilitation of cancer cell invasion and metastasis.

### Natural killer cells

Natural killer (NK) cells are intrinsic immune cells that have the rapid capacity to recognize and clear infected cells. A growing number of experimental and clinical studies have shown that NK cell-based antitumor immunotherapy is highly promising (43). NK cells play an essential position in controlling the metastasis and proliferation of cancer cells. NK cells differentiate tumor stem cells or undifferentiated tumors by secreting or membrane-bound IFN-γ and TNF-α and prevent tumor growth by modifying the tumor microenvironment (44). In addition, as documented in multiple experiments, NK cells can distinguish abnormal cells from healthy ones, leading to more specific antitumor cytotoxicity and reducing off-target complications (45).

Mounting evidence in human cancer indicates that NK cell frequency, infiltration, and function improve patient survival (46, 47).

Huyan T et al. (21) researched five polysaccharides derived from plants or fungi including *G. frondosa* polysaccharide (GFP), lentinan (LNT), *G. lucidum* polysaccharide (GLP), *Lycium barbarum* polysaccharide (LBP), and yeast glucan (YG). They found that at a concentration of 100 mg/L, these polysaccharides could have an apparent positive effect on NK cell proliferation, and the three polysaccharides, GLP, LBP and LNT, increased the cytotoxicity of NK cells after 48 h of treatment.

GLP and LBP treatment could significantly upregulate the mRNA expression of IFN-γ, perforin and granzyme-B, while the expression of the surface activating receptor NKp30 was significantly u-regulated after GLP, LBP, and LNT treatment, which can promote NK cell secretion of TNF-α and IFN-γ when stimulated by its ligand, NKp30L. In addition, LBP and GLP treatment enhanced NK cell function under simulated microgravity (SMG) conditions by restoring the expression of the activating receptor NKG2D and reducing early apoptosis and late apoptosis/necrosis.

Another traditional Chinese medicine Danggui (*Angelica sinensis* (Oliv.) Diels.) can target the liver, and one of its essences lies in the existence of polysaccharide components, *A. sinensis* polysaccharides (ASP). Liu X et al. (48) synthesized amphiphilic polymer micelles contained ASP, achieving a hypoxic response to drug release, and found enhanced cell uptake and effectively improved proliferation inhibitory activity of HepG2 hepatocellular carcinoma cancer cells.

Yupingfeng (YPF) is a traditional formula in Chinese medicine, consisting of Huangqi (*Astragalus membranaceus* (Fisch.) Bge.), Baizhu (*Atractylodes macrocephala* Koidz.), and Fangfeng (*Saposhnikovia divaricata* (Turcz.) Schischk.*)*, which has Qi nourishing properties. In non-small cell lung cancer (NSCLC) therapy, compared with the untreated control, YPF (22) treatment significantly inhibited the growth of Lewis lung cancer (LLC) cells and prolonged the median survival time for LLC-bearing mice. Furthermore, the results of the flow cytometry analysis revealed that YPF treatment increased NK cell tumor infiltration and the NK cell population in spleen.

By isolating splenic cells and evaluating their cytotoxicity through a calcein-release assay, YPF treatment was demonstrated to enhance NK cell-mediated killing activity. After using anti-NK1.1 antibody PK136, the tumor suppressive effect of YPF was reversed, which implied that the suppression of tumor growth by YPF was NK cell-dependent. YPF probably inhibits cancer development by increasing IL-12 expression to promote NK cell activation while decreasing TGF-β, IDO, and IL-10.

## Regulation of the adaptive immune system

### CD4^+^T cells

CD4^+^ T cells contain two diverse subsets of helper T cells, namely, Th1 cells, which produce TNF-α, IFN-γ, IL-2, and IL-12 to mediate antitumor effects, and Th2 cells, which generate IL-4 and IL-10 and foster tumor growth by suppressing the host immune system (49).

According to clinical data, cancer patients suffer from a Th1/Th2 imbalance, with increased cytokine release from Th2 cells. Patients with Th1-led responses have higher survival rates and lower cancer recurrence rates. Consequently, the development of new therapeutic strategies to modify the Th1/Th2 balance may assist in the control of cancer (50).

Saikosaponin A (SSa) is an effective ingredient extracted from the radicals of the root of *Bupleurum chinense* DC. or *Bupleurum scorzonerifolium* Willd. Tumor volume was significantly reduced in the tumor-bearing rats treated with SSa (23) compared with those in a control group. After treatment with SSa, there was also weak positive Ki-67 staining, which is a proliferative biomarker of tumor cells and is associated with a poor prognosis.

Detection of signature cytokines revealed that SSa treatment significantly promoted IFN-γ and IL-12 secretion and inhibited IL-4 and IL-10 secretion, indicating that SSa could induce Th1/Th2 shifting into Th1 cell response. Compared with the control group, the SSa group showed significantly increased protein expression levels of IL-12, protein and mRNA expression levels of IL-12R, mRNA expression levels of STAT4, and protein expression levels of pSTAT4, which indicated that SSa could activate the IL-12/STAT4 pathway.

Rougui (*Cinnamomum cassia* Presl) is a commonly used herb that disperses Cold and relieves pain. It is often used in Chinese medicine to treat impotence, lumbago and menstrual pain.

High-energy ion radiation (IR) has been shown to induce immune damage in clinical applications, investigating the antitumor immune response of single low-dose total body irradiation (SLTBI), Zheng X et al. (24) experimented.

After SLTBI treatment in 57BL/6 mice with melanoma model, both the cell counts and the frequencies of total T cells (CD3^+^) and CD4^+^ and CD8^+^ T cells from the spleen were significantly decreased within 3 to 5 days, and CD3^+^CD4^+^T cell restoration started from day 8 and returned to normal levels 10 days after exposure, which showed that the reconstitution of CD4^+^ T-helper cells was delayed after SLTBI. Although T-cell numbers increased at 10 days after SLTBI, IFN-γ production released by Th1 or by Tc1 was still at a lower level in irradiated mice than in control mice, whereas the proportions of Th2 (CD3^+^CD4^+^IL-4^+^), Th17 (CD3^+^CD4^+^IL-17A^+^), and Treg (CD4^+^CD25^+^Foxp3^+^) cells were significantly increased in the IR group.

Cinnamon-treated up-regulated Th1 and Tc1 cells were significant, whereas Th17 and Treg cells were down-regulated. After administration of cinnamon, T-bet expression increased and expression of Foxp3 decreased, while the transcription levels of RORgt and GATA-3 were not changed significantly. The result showed that cinnamon modulates specific signaling of distinct T-cell subsets, primarily activating Th1 and inhibiting Treg.

Banxia(*Pinellia ternata* (Thunb.) Breit.), a traditional Chinese herbal medicine, show a function as reducing swelling and drying Dampness. Huang H (25) et al. used a novel *Pinellia ternata* lipid-soluble extract (PE) to treat HPV(+) tumors *in vivo* and *in vitro*.

They co-cultured naïve CD4+ T cells and DCs, and treated with PE solution, founding that PE treatment significantly upregulated beta-catenin and c-myc levels *in vitro.*

In C57BL/6 mice burdened with the HPV+TC-1 tumor by PE treatment, flow cytometry analysis resulted in the percentage of CD4+ T cells in peripheral blood mononuclear cells (PBMCs) and tumor-infiltrating lymphocyte (TIL) under PE-low treatment and in TIL under PE-high treatment was obviously increased. With PE-low treatment, T-bet expression was expressed more highly in the PBMCs and TIL of mice bearing HPV+TC-1 tumors than in the solvent control group. The IFN-γ production level was also significantly increased in PBMCs compared with TIL, which showed that Th1 cells increased significantly. Additionally, they found that GATA3 and IL-14 had a lower expression in the PBMCs and TIL of mice after PE-low treatment, indicating the downregulation of Th2.

### CD8^+^ T cells

The main function of activated CD8^+^ T cells (cytotoxic T cells) is to secrete perforin, TNF-α, and FASL, specifically killing target cells. Their antitumor function is mainly dependent on two factors: the differentiation of T cells and the infiltration of CD8^+^ T cells, i.e., the transport of CD8^+^ T cells into the tumor microenvironment, where they secrete cytotoxic factors and exert tumor lysis (51). Increased levels of CD8^+^ T cells in the tumor microenvironment have been demonstrated to be correlated with antitumor effects in a wide range of cancer types, demonstrating a correlation between increased levels of CD8^+^ T cells and cancer prognosis.

Biejiajian pill (BJJP) is a classical formula in the “*golden chamber synopsis*”, consisting of 23 components. In herbal medicine, it is extensively used in the therapy of liver cancer. Yang X et al. (26) compared the antitumor effects of BJJP in immunodeficient BALB/c-nu/nu and immunocompetent BALB/c mice using the H22 tumor cell model. They found that after treatment with BJJP, the tumor weights and volumes in the immunocompetent BALB/c mice were notably lower than those in the immunodeficient BALB/c-nu/nu mice and that the BJJP-treated immunocompetent BALB/c mice had the highest rate of apoptosis, suggesting BJJP can promote antitumor immunity in HCC. Immunofluorescence or fluorescence-activated cell sorting showed that CD8^+^ T cell infiltration after treatment with BJJP and BJJP treatment also promoted the effector function of CD8^+^ T cells by significantly increasing the expression of TNF-α and IFN-γ in tumor-infiltrating CD8^+^ T cells.

Chemokines and chemokine receptors are necessary for the recruitment of CD8^+^ T cells into tumors, which were identified to regulate the migration of CD8^+^ T cells in previous studies. Compared with the control groups, there was a significant increase in the expression of CCL5 in the subcutaneous tumors of mice in the BJJP group. When co-stained CCL5 with CD8^+^ T cells, fields with higher CCL5 expression showed more CD8^+^ T cell infiltration, and higher levels of serum CCL5 were observed in the BJJP-treated group.

Spleen cells from BABL/c mice were co-cultured with GFP^+^H22 cells, and the results revealed a significant increase in the number of migrating CD8^+^ T cells in the BJJP-treated group, and migration occurred in a dose-dependent manner *in vitro*. In addition, in the BJJP-treated group, there was a 2- and 3-fold increase in the mRNA and protein expression levels of CCL5.

BJJP is a compounded formula containing many herbs, some of which have been developed at the nanoparticle level.

Zhang M (52) created nanoparticles from ginger and reassembled their lipids into ginger-derived nanovectors (GDNVs), were capable of loading Dox with high efficiency, and showed a better pH-dependent drug-release profile. Furthermore, Dox-GDNVs activated caspase-3/7 and exerted apoptotic effects in Colon-26 and HT-29 cells. In BALB/c nu/nu female mice bearing Colon26 subcutaneous xenografts, treatment with Dox-FA-GDNVs downregulated Ki67 expression, resulting in a significant decrease in the number of cancerous cells compared with saline, free Dox, and FA-GDNVs groups, showing effective in targeting tumors and reducing their volume.

Haggag YA (53) developed a novel PEGylated-PLGA polymeric nanocapsules (NCs) for HK delivery to breast tumor-bearing mice. HK-loaded NCs achieved significant cytotoxic action compared to free HK due to successful HK delivery to the subcellular site of action2 meanwhile preserving its anti-cancer activity after formulation.

HK-loaded NCs showed a significant increase in caspase-3 level and decrease in VEGF-1 level in tumor tissue compared to control animals and the free HK treated group, which confirmed HK-loaded NCs had a potent antiangiogenic and apoptotic activity compared to free drug.

Gastrodin (GAS) is an organic compound extracted from TCM Tianma (*Gastrodia elata* Bl.), which is used to treat strokes, convulsions, vertigo, headaches, and numbness in the limbs. *In vitro*, GAS increased CD80, CD86 and MHCI, which are APC activation markers, in DC2.4 cells after treatment compared with the negative control. *In vivo*, GAS treatment in mice also stimulated the activity of APCs. Intracellular staining of splenocytes and flowcytometric analysis showed that the expression levels of IL-12 and TNF-α were upregulated.

The expression levels of perforin and TNF-α were increased by GAS + cell treatment compared with control group, and cytotoxic lymphocytes were significantly increased in mice immunized with GAS, which suggested that GAS can promote the responses of CD8^+^ T cells by enhancing T-cell proliferation and inflammation cytokine expression. Liu Z et al. (27)demonstrated the immunogenicity of GAS as an adjuvant in an anti-melanin vaccine when they found that GAS enhanced the activation of antigen-presenting cells (APCs) - both *in vivo* and *in vitro*.

Wang J et al. (54) developed a new nano formulation encapsulated with GAS, and GAS was released and delivered into the brain, where its scavenged the oxygen free radicals produced from cavitation, providing an approach to monitor and inhibit cell apoptotic events.

### Regulatory T cells

Regulatory T cells (Tregs) are a subset of CD4^+^ T cells that control autoimmune responses and maintain immune homeostasis. During tumor development, tumor cells and macrophages in the regional tumor microenvironment secrete chemokines that recruit Tregs from peripheral blood into the tumor, allowing Tregs to escape from the host immune system due to the immunosuppressive function (55).

Higher expression of Tregs tends to suggest decreased patient survival. In the field of tumor immunotherapy, there is an acute demand to reduce the number of Tregs to increase antitumor immune response (56).

Oridonin is an active ingredient isolated and extracted from the herb Donglingcao (*Rabdosia rubescens* (Hemsl.) Hara), which is known for its ability to clear heat, promote blood circulation, and relieve pain. Guo J (28) injected 4T1 cells subcutaneously into WT BALB/c mice to assess the effect of oridonin on the growth of 4T1 murine triple-negative breast cancer (TNBC) *in vivo* and found that oridonin treatment suppressed tumor progression compared with control mice. TILs from oridonin-treated mice had a significantly reduced number of Tregs, but the proportion of CD8^+^ T cells was increased. In addition, the spleens of the oridonin-treated mice also demonstrated a decreased percentage of Tregs and an enhanced level of IFN-γ^+^CD8^+^ T cells. Similarly, *in vitro*, oridonin decreased the number of Tregs in a dose-dependent manner and attenuated the induction of Foxp3^+^CD4^+^ T cells.

Oridonin-treated Tregs had lower expression of CTLA4 and CD69 and lower protein secretion levels of TGF-β1 and IL-10 than control Tregs. Tregs treated with oridonin displayed a diminished ability to suppress the proliferation of CD8^+^ T cells, and the phosphorylation of Smad2 and Smad3 was notably decreased in oridonin-treated Tregs. Furthermore, oridonin inhibited the protein levels of TGF-βRI and TGF-RβII, and when utilizing TGF-βreceptor inhibitor SB431542, oridonin could not further suppress Treg differentiation, indicating that oridonin represses Treg polarization by decreasing the TGF-β receptor.

In addition, in treating 4T1 tumors in WT BALB/c mice, therapy with oridonin and anti-PD-1 resulted in superior tumor regression compared with treatment with either oridonin or anti-PD-1 alone. Oridonin+PD-1 treatment resulted in more CD8^+^ T TILs and increased numbers of IFN-γ^+^CD8^+^ T cells and granzyme B^+^CD8^+^ T cells, showing that oridonin improves the antitumor activity of anti-PD-1 in breast cancer.

Yi-Yi-Fu-Zi-Bai-Jiang-San (YYFZBJS) is from *the Golden Chamber* and is commonly used in traditional Chinese medicine to treat gastrointestinal disorders. It is composed of three herbs: Yi-Yi-ren (*Coix lacryma-jobi L.var.ma-yuen* (Roman.) Stapf), Fuzi (*Aconitum carmichaelii* Debx.), Baijiangcao (*Patrinia scabiosaefolia* Fisch. ex Link., P. villosa (Thunb.) Juss.), considered in the theoretical system of Chinese medicine to have the effect of reducing swelling and draining pus, and is used in the treatment of *Changyong*, known in modern medicine as appendicitis.

Sui H (29) et al. gavaged germ-free *Apc^Min/+^* mice with stool from healthy controls and YYFZBJS volunteers, and discovered that *Apc^Min/+^* mice treated with YYFZBJS carried fewer adenomas both in the small and large intestine, and animal weight and hepatorenal toxicity among treatment groups during the experiment, confirming the safety of these herbs. The proliferation markers, nuclear expression levels of Ki67 and PCNA, and BrdU reactivity in intestinal polyp epithelia were reduced after YYFZBJS treatment.

Compared to normal control mice, *Apc^Min/+^* mice secreted higher levels of inflammatory cytokines/chemokines, including IL-17, Eotaxin-2, Leptin and PF4, and YYFZBJS treatment downgraded IL-17, and IL-10 in myeloid precursor differentiation, additionally, IL-6, CCXL13 and IL-10 also had a lower level expression than the untreated group. Further results of ELISA showed that the expression IL-17A and TNF-α were upregulated by YYFZBJS treatment, which suggested that YYFZBJS blocked tumor progression in CRC murine model possibly *via* inhibiting the accumulation of Treg cells in immune organs, and in tumor microenvironment.

To determine whether the presence of gut commensal bacteria affects regulatory T cells, *in vitro* experiments showed that YYFZBJS extract treatment could inhibited the increased-Foxp3-mRNA expression when the *enterotoxigenic Bacteroides fragilis* (ETBF) were co-incubated with CD25^+^/CD4^+^ T cells which isolated from the spleens of *Apc^Min/+^* mice. Compared with the ETBF primed Treg group, the proliferation of MC-38 cells were decreased after treated with YYFZBJS extract and ETBF primed Treg. Western blotting results showed that YYFZBJS could reverse the distribution of β-catenin in the nucleus, which is ETBF primed Treg significantly increased.

## Regulation of tumor-associated inflammation

Inflammation is part of the innate immune response to danger signals, tissue disruption, and/or infection. Properly terminated, inflammation is beneficial; however, chronic inflammation increases cancer risk and has a great effect on the composition of the tumor microenvironment and particularly on the plasticity of both tumor and stromal cells (57).

Extensive epidemiological studies have confirmed that chronic inflammation predisposes people to various cancers. Cancer-related inflammation impacts many aspects of malignancy, including tumor cell proliferation, angiogenesis, metastasis, and tumor response to chemotherapeutic agents and hormones (58, 59).

Tumor-associated inflammation, which entails intricate interactions between epithelial and stromal cells, can in some cases lead to epigenetic alterations that drive malignant progression and even initiate tumorigenesis. Chronic inflammation results in the production of growth factors that support the development of newly emergent tumors (60).

The continuous production of various cytokines, chemokines and growth factors within the tumor microenvironment supports cancer cell proliferation, evolution, and survival as well as tumor vascularization and immune dysregulation, all of which contribute to tumor progression, invasion, metastasis and therapy resistance.

The active components of traditional Chinese herbs can modulate the expression of some cytokines, thus affecting inflammatory inflammasome and nuclear factors to exert a regulatory effect on tumor-associated inflammation.

### NLRP3 inflammasome

The NLRP3 inflammasome can be activated by pathogen-associated molecular patterns (PAMPs) or damage-associated molecular patterns (DAMPs), causing caspase-1 cleavage and maturation of IL-1β production. Concurrently, NLRP3 can be activated by LPS in the presence of PAMPs, subsequently exacerbating cancer progression (61).

Studies have shown that LPS-induced pro-inflammatory cytokine expression in NSCLC can be associated with the clinical prognosis of metastatic NSCLC, where Gram-negative bacteria, the primary pathogens, adversely affect NSCLC through the production of LPS (62). Targeting LPS-induced inflammation can effectively ameliorate the proliferation and migration of NSCLC cells *in vivo*.

Hongjingtian (*Rhodiola crenulata* (Hook. f. et Thoms) H. Ohba.) is a traditional Chinese medicine with properties of Qi benefit, blood activation, and asthma calming, and salidroside (30) (SAL) is an active extract of Hongjingtian.

Coincubation with SAL for 24 h and 48 h suppressed the LPS-induced increase in A549 cell proliferation in a concentration-dependent manner, and SAL treatment inhibits LPS-induced migration.

A549 cells exposed to LPS for 4–24 h showed lower levels of AMPK phosphorylation and higher levels of NLRP3, caspase-1, and IL-1β than untreated cells, and LPS-treated cells also exhibited impaired AMPK activity and enhanced NLRP3 inflammasome activation. In contrast, cells treated with SAL following LPS-induction showed higher levels of phosphorylated AMPK.

In addition, when AMPK was inhibited by compound C, LPS and SAL cotreated cells showed increased levels of NLRP3, pro-caspase-1, caspase-1, pro-IL-1β, and IL-1β, which suggests that SAL suppresses LPS-induced activation of the ROS/NLRP3 inflammasome axis in A549 cells through its effects on AMPK. The efficacy of SAL in NSCLC has been demonstrated, particularly in cases of concomitant bacterial infection.

Ginkgolide B (GKB) is the main active ingredient of Yinxingye (*Ginkgo biloba* L.) extract, which activates blood circulation, resolves blood stasis, relieves pain, and calms asthma.

Wang X et al. (31) treated H1975 and A549 cells with GKB, and GKB time-dependently inhibited tumor cell proliferation. The expression of beclin-1, p62, and LC3B proteins was strongly induced in response to GKB treatment in lung tumor cells in a time-dependent manner by western blot analysis. Furthermore, the expression of PCNA and Bcl-2 decreased in a time-dependent manner in GKB-treated A549 and H1975 cells. After using the selective autophagy inhibitor 3-MA to block autophagy, GKB decreased the number of invasive cells *in vitro*, and the treatment interfered with the GKB-induced decreased number of cell colonies.

Treatment of H1975 cells with GKB for 24 h significantly reduced NLRP3 expression, leading to pro-IL-1β and pro-IL-18 being cleaved to create the mature bioactive form. Furthermore, after using autophagy inhibitor 3-MA, the GKB-induced inhibition of IL-1β and IL-18 expression were reversed. These results showed that GKB-induced inhibition of the NLRP3 inflammasome can be reversed by beclin-1 knockdown in H1975 cells.

### NF-κB

The nuclear factor kappa B (NF-kB) family consists of transcription factors that act as an integral part of a network that determines its pattern of expression on other genes, regulating a variety of functions, including inflammation, immune responses, cell proliferation, and survival (63). The NF-kB pathway contributes to the progression and metastasis of several cancer types, including breast cancer (64), prostate cancer (65), and nasopharyngeal carcinoma.

Chemokines and their receptors (CXCR4 and CCR7) are critical in determining tumor cell metastasis. The expression of CCR7 and CXCR4 mRNA in LPS-induced MCF-7 and MDAMB-231 breast cancer cells was significantly reduced by treatment with puerarin, a flavonoid isolated from Gegen (*Pueraria lobata* (Willd.) Ohwi.), thereby significantly dampening the adhesion of cells to extracellular matrix components. Liu X et al. (32) found that puerarin inhibited LPS-stimulated migration of MCF-7 and MDA-MB-231 cells and dose-dependently reduced the mRNA expression and protein levels of CCR7 and CXCR4 in LPS-induced MCF-7 and MDA-MB-231 cells.

Co-treatment with puerarin inhibited LPS-induced cell invasion in breast cancer, and the mRNA and protein expression levels of MMP-2 and MMP-9 were reversed by puerarin treatment. In addition, compared with the LPS group, puerarin significantly reduced cell attachment to collagen I, collagen IV, and fibronectin. Furthermore, the mRNA and protein expression levels of the adhesive molecules VCAM-1 and ICAM-1 were obviously reduced in the puerarin group.

Prestimulation with LPS triggered phosphorylation of p65, and this effect was reversed by co-treatment with puerarin in MCF-7 and MDA-MB-231 cells. Puerarin also suppressed the phosphorylation of IκBα and Erk1/2 in breast cancer cells, which indicated that puerarin disrupted NF-κB signaling *via* suppression of p65 nuclear translocation and IκBα phosphorylation.

Xu H et al. (66) developed a novel puerarin nanoemulsion (nanoPue) that facilitated the chemotherapy effects of nano-paclitaxel in a desmoplastic TNBC model and increased intra-tumoral infiltration of cytotoxic T cells to synergize PD-L1 blockade therapy in this TNBC model.

Cisplatin chemotherapy is a standard clinical option for the treatment of NSCLC (67). Hypoxia is prevalent in multiple solid tumors and is closely related to tumor proliferation, metastasis, clinical stage, and treatment outcome as well as patient prognosis. Under hypoxic conditions, the NF-κB pathway is activated, tumor proliferation and metastasis are enhanced, human NSCLC cells are less sensitive to cisplatin, and the efficacy of cisplatin chemotherapy is compromised.

Wang J et al. (68) found that CoCl2-induced hypoxia decreased the sensitivity of NSCLC cells to cisplatin and significantly reduced the colony formation rate and total apoptosis rate of cisplatin-treated cells compared with cells in normoxia. They demonstrated that expression of the pro-apoptotic proteins (caspase-3, -8, and -9 and Bax) and apoptosis markers (PARP) were lower in hypoxia than in normoxia with cisplatin; furthermore, anti-apoptotic proteins (Bcl-2 and survivin) were significantly increased. In addition, hypoxia significantly activated the expression of HIF-1α, Snail, N-cadherin, and vimentin, while it decreased the expression of E-cadherin. The expression of SOX2, NANOG, OCT4, and CD44 were upregulated in hypoxia, which indicated that NSCLC cells undergo EMT and obtain stemness in hypoxia.

Under hypoxic conditions, there was increased expression of p-p65, p65, p-IKK, and IKK and downregulated expression of EMT-related molecules including Snail, N-cadherin, and vimentin, while E-cadherin protein expression was upregulated. In addition, the stemness-related molecules SOX2, NANOG, OCT4, and CD44 were downregulated. The results suggested that inhibition of the NF-κB pathway could reverse hypoxia-mediated EMT and stemness.

Compound 20 (R)-ginsenoside (Rg3) is an active monomer extracted from the TCM herb Renshen (*Panax ginseng* C. A. Mey.), which is considered to be a tonic for Qi and nourishes Yin in traditional Chinese medicine and has been shown to inhibit cancer cells. Some studies indicate that Rg3 can suppress EMT *via* suppressing Notch-Hes1-EMT signaling (33).

Rg3 treatment of hypoxic NSCLC cells decreased the expression of p-p65 and p65 in a concentration- and time-dependent manner, and NF-κB DNA binding in the Rg3 + cisplatin group was less than that of the two drugs used individually in hypoxic cells. The expression levels of p-p65 and p65 in nuclear protein extracts were the lowest in the Rg3 + cisplatin-treated group in hypoxia, and the expression levels of p-p65, p65, p-IKK, and IKK in total protein extracts were lowest in the cells treated with Rg3 + cisplatin. Thus, Rg3 inhibited the activation of the NF-κB signaling pathway under hypoxic conditions, and Rg3 + cisplatin increased this inhibitory effect.

They found that hypoxic NSCLC cells treated with Rg3 + cisplatin had significantly decreased cell viability compared with treatment with Rg3 or cisplatin individually, and the Rg3 + cisplatin-treated cells had a significantly lower colony formation rate than cells treated with cisplatin or Rg3 alone in hypoxia. Furthermore, the flow cytometry results showed that Rg3 + cisplatin treatment led to an increase in the total apoptosis rate compared with treatment with cisplatin or Rg3 alone in hypoxic cells. The Rg3 + cisplatin group showed higher expression of pro-apoptotic proteins (caspase-3, -8, -9 and Bax) and apoptosis markers (PARP) than the individually treated groups, and the expression of anti-apoptotic proteins (Bcl-2 and survivin) showed the opposite trend.

Cao M (69) isolated and characterized from *Panax ginseng* C. A. Mey., and developed a novel EVs-liked ginseng-derived nanoparticles (GDNPs) with superb stability and biocompatibility. After treatment with GDNPs, the expression of CD80, CD86, MHC-II, TLR2/4, IL-6 and TNF-α was up-regulated, which revealed that GDNP exposure significantly induced M1-related markers. Moreover, GDNPs treatment increased the production of M1-related cytokines and chemokines, such as CCL5, IL-6, MCP-1, TNF-α IL-1α and IL-12, altering the M2-like polarization of macrophages *in vitro*. Treatment with GDNP-stimulated macrophages significantly increased apoptosis of B16F10 melanoma cells and caspase- 3/7 expression compared to treatment with a control medium.

## Active ingredient nanotechnology combination with herbal medicine for tumor immune modulation

In recent years, nanotechnology has been applied in various fields of biomedicine, with the emergence of new drug delivery systems based on nano-formulations that are expected to overcome the current limitations of tumor therapy, offering the advantages of increased efficacy, improved absorption, and reduced adverse effects (70). This paper focuses on the antitumor effects of the active ingredients of TCM in combination with liposomes, carbon nanomaterials, and metal nanoparticles.

### Liposomes

Zhu J et al. (71) developed a novel nano-lipid carrier formulation, NCNLC, using coix seed oil (CSO) extracted from the TCM Yiyiren (*Coix lacryma-jobi* L.var.ma-yuen (Roman.) Stapf) as a functional liquid lipid, loaded with naringin (NG), a model anti-HCC drug, by ultrasonic melt emulsification. Drug release was promoted when CSO acted as a liquid lipid. However, when comparing nanostructured lipid carrier (NLC) prepared from free NG, neodecanoic triglyceride (NDNLC), and oleic acid (NONLC), NCNLC was more effective in antitumor proliferation and promoting apoptosis among tumor cells. In particular, NG + CSO upregulated the expression of IL-6 and IL-10 in the serum of tumor-bearing mice and significantly increased the spleen index, suggesting that the combination of the two *via* nanotechnology could enhance the antitumor immune effect.

Doxorubicin (DOX) is one of the most widespread antitumor drugs used in prostate cancer (pCa), but its side effects are unavoidable. Tanshinone is an active extract of the Chinese herb Danshen (*Salvia miltiorrhiza* Bge.*)*, which has now been shown to possess wide-ranging anticancer effects.

Prostate-specific membrane antigen (PSMA) is a cell surface protein that is a recognized tumor marker, including for pCa, and is an established target for targeted drug delivery. Sun G et al. (72) combined pH-sensitive nanoparticles and prepared lipid nanoparticles loaded with DOX and TAN by emulsification and solvent diffusion to form a P-N-DOX/TAN nanodelivery system. This system can be adapted to the acidic environment of tumor tissue (pH 6.5) and can be released more entirely at the tumor area. Due to the modification of the PSMA-targeting ligands, the LNCaP tumor cells showed a high degree of absorption of P-N-DOX/TAN, which facilitated the accumulation of the drug *in vivo*. The drug-loaded nanoparticles were more stable than those in the drug-free group and had a superior effect on apoptosis, showing a potent inhibitory effect on cancer cells.

### Carbon nanomaterials

Pishuang is a natural product of TCM, and As2O3 (ATO), the main compound of *arsenic*, can be used to remedy a variety of cancers such as breast cancer (73) and pCa (74). Several limitations exist in its clinical application, such as poor pharmacokinetics and unacceptable systemic toxicity. There are also severe limitations to the use of ATO in the treatment of gliomas, as its poor ability to penetrate the blood-brain barrier makes it less effective.

RGDArg-Gly-Asp (RGDyC) acts as a ligand that preferentially binds to receptors on the surface of leukocytes, bringing NPs into the brain for targeted delivery to tumor tissue. Simultaneously, polyamidoamines (PAMAMs) may be useful carriers that can add to the biodistribution of the drug and potentially augment the targeting effect. Lu Y et al. (75) proposed a targeted drug delivery system based on RGDyC and PEG co-modified PAMAM (RGDyC-mpeg-PAMAM/ATO). RGDyC-mPEG-PAMAM/ATO treatment significantly inhibited the growth of glioma in Wistar rats by repressing cell proliferation and inducing apoptosis while reducing the toxicity of ATO, prolonging the survival time of rats and enhancing the antitumor effects of ATO.

### Metal and/or metal oxide nanoparticles

With their excellent biocompatibility and autologous liver targeting, iron-based NPs show significant advantages as multifunctional carriers for chemotherapeutic drug delivery. The ginsenoside Rg3 is a potent extract of the TCM *ginseng*, which has been demonstrated to induce cellular autophagy and sensitize liver cancer cells to adriamycin (76).

Ren Z et al. (77) developed a novel nanomedicine (NpRg3) by synthesizing and coupling Fe@Fe3O4 NPs with the ginsenoside Rg3. NpRg3 treatment significantly prolonged the survival time of tumor-bearing mice, improved biochemical parameters such as ALT and AST, as well as reduced tumor proliferation and aggressiveness.

Rg3 acts as a cancer suppressor by regulating VEGF-dependent tumor angiogenesis, while the long-term circulation of iron-based NpRg3 could have a therapeutic effect on circulating tumor cells by activating immune cells in the blood. In comparison to the other groups, no lung metastases from HCC were observed in the NpRg3-treated group, and lung metastases from HCC were significantly inhibited, demonstrating its superior antitumor metastatic effect.

## Dilemmas of Chinese herbal medicine applications

Chinese herbal medicine has long been used traditionally to treat patients with cancer. In particular, combined TCM and Western medicine are usually used for cancer patients who have undergone surgical resection in conjunction with radiotherapy and chemotherapy as a maintenance or alternative treatment. Nonetheless, as cases of adverse drug reactions (ADRs) increase with the popularity of Chinese herbal medicine, attention should also be drawn to the adverse effects caused by herbal medicine.

### Toxicity

Madouling (*Aristolochia debilis* Sieb. et Zucc) is an herb that is believed in TCM theory to be effective in clearing heat and relieving coughs and asthma. Aristolochic acids (AASs) are potent carcinogens and are extremely nephrotoxic (78). Excessive intake of AASs is a major cause of aristolochic acid nephropathy (AAN).

The herb Wutou (*Aconitum carmichaeli* Debeaux) is used to treat impotence, abdominal pain, edema and joint pain, and it is also known as a poison because of the presence of aconitine, a highly toxic alkaloid. Fresh Wutou must be fried, steamed, or fumigated to reduce its toxicity for oral administration. Aconitine’s toxic effects inhibit the myocardial tricarboxylic acid cycle and oxidative respiratory chain phosphorylation, resulting in impaired myocardial aerobic metabolism and cardiac dysfunction, causing severe cardiotoxicity (79).

### Adverse drug reactions

Generally speaking, except for drugs that have been demonstrated to be poisonous, the side effects of oral Chinese medicine are not obvious; the main side effects exist in Chinese medicine injections.

ADRs primarily involve cutaneous and appendage injuries, general body disturbances, and autonomic nervous system disturbances, including rash, pruritus, anaphylactic reactions, and palpitations. Hypersensitivity shock and anaphylactic-like reactions are among the rarest serious ADRs associated with herbal injections, posing an increased threat to patient safety and even death (80).

## Discussion

The active ingredients in TCM are very complex and contain a variety of different types of compounds. In the theory of TCM, polysaccharides and saponins, such as *G. lucidum* polysaccharides and ginsenosides, are usually found in medicines that can help make Qi and blood flow better.

Modern research has also shown that ginsenosides can help the immune system switch from a Th1 to a Th2 phenotype, release interferons, facilitate dendritic cell growth, and promote immunomodulatory effects in different types of cancer (81) (82).

Flavonoids can be found in the human diet in fruits, vegetables, legumes, tea, dark chocolate, etc. Some specific parts of these foods are richer in flavonoids than others, such as the peels of certain fruits (83). Flavonoids are generally considered to be associated with intestinal metabolism (84), and the herbs that are considered to promote digestion in TCM theory also basically contain flavonoids, such as Shanzha (*Crataegus pinnatifida* Bge. var. major N. E. Br., *Crataegus pinnatifida* Bge.). Flavonoids are generally considered to have anti-inflammatory properties; for example, one flavonoid, quercetin, is able to achieve anti-inflammatory effects by inhibiting the NF-κB pathway (85).

Alkaloids are the main active ingredients of another group of herbs, generally contained in medicines that are considered to clear Heat in TCM theory. For instance, berberine has been reported to possess anticancer activities. Among the various cellular targets of berberine is AMPK, which regulates tumor progression and metastasis. Berberine activated AMPK in human colon cancer cell lines. Notably, berberine-induced activation of AMPK, reduced integrin β1 protein levels, and decreased the phosphorylation of integrin β1 signaling targets (81).

In addition to the above, there are many unexplored and under-explained active ingredients in Chinese herbal medicine that remain to be discovered through further experiments. (The effects of some of the actively ingredients commonly used in herbal medicine are listed in **Table 4**).

**Table 4:** A list of active ingredients of Chinese herbal medicines proven to have anti-cancer immune effects.

Active ingredient	Immunomodulatory effects	Reference
**Astragaloside IV**	↓IL-13 and IL-4, ↓M2 macrophages	(82)
**Astragalus polysaccharides**	↑dendritic cells (DCs) ↑T-cell proliferation	(86)
	↑CD4^+^, CD25^+^Treg	(87)
	↑TLR4 signaling pathway,↑IFN-γ, IL-2 and IL-4 ↓genes PFP, GraB, Fas L and Fas, ↓TGF-β	(88)
**Achyranthes bidentata polysaccharides**	↑DCs	(89)
**Cordyceps sinensis**	↓LPS,↑Th1→Th2	(90)
**Lupeol**	↑NK cells, ↑PI3K/Akt and Wnt/β-catenin signaling pathways	(91)
**Zanthoxylum piperitum DC**	↑perforin, ↑granzyme B, ↑NK cells	(92)
**Ganoderma**	↑ NK cells	(93)
Ganoderma **atrum polysaccharide**	↑Th1 cytokine production ↑CTL and NK cell cytotoxic activity ↑caspase-3 and caspase-9	(93)
**Ganoderma lucidum polysaccharides**	↓Tregs,↓Notch1 and FoxP3 expression ↑IL-2,↑miR-125b expression	(94)
Dendrobium huoshanense **polysaccharides**	↑Th1, Th2 ↑inflammatory cytokines and chemokines	(95)
**Icariin**	↑CD8+ T cells,↑IFN-γ ↓MDSCs,↓L-10, IL-6 and TNF-α	(96)
**Asparagus polysaccharide**	↓MDSCs, Bcl-2 ↑Bax, caspase-9	(97)
**Ginsenoside**	↑DCs, ↑Th1→Th2	(98)
**Ginsenoside Rg3**	↑Th1, ↑IL-2 and IFN-γ	(99)
**Radix Glycyrrhizae polysaccharide (GP)**	↓Treg,↓Foxp3 and IL-10 mRNA expression ↓IL-10 and TGF-β	(100)

*AS-IV, astragaloside IV, APS, astragalus polysaccharides, ABPS, Achyranthes bidentata polysaccharide, CS, Cordyceps sinensis, ZPDC, zanthoxylum piperitum DC, PSG-1, Ganoderma atrum polysaccharide, GLPS, Ganoderma lucidum polysaccharides, DH-PS, dendrobium huoshanense polysaccharide, ICA, icariin, AP, asparagus polysaccharide, GP, Glycyrrhizae polysaccharide. ↑ implies an increase in the relevant level and ↓ implies a decrease in the relevant level.

## Conclusion

The effect of Chinese medicine on the immune system can be sophisticated. Chinese herbal medicines are rich in a variety of chemical components, including polysaccharides, adenosine, and alkaloids. Different herbs used individually or different combinations of different herbs and various active ingredients in multiple preparations can also yield distinct immunomodulatory outcomes.

TCM, particularly herbal medicine, enjoys a distinguished history of clinical application in China and has enormous potential for development. Thousands of years of experience in clinical practice have provided the diagnostic basis for the medical treatment of diseases, but the chemical composition of some traditional antitumor Chinese medicines in clinical use has not yet been entirely identified. Therefore, further in-depth research is warranted. Despite the fact that research on the mechanisms of antitumor action of Chinese medicine is still in an early phase, it is still a worthwhile consideration to use Chinese medicine as a promising direction for antitumor immunotherapy.

There are limitations in the application of TCM in antitumor therapy; however, further research is required to identify the valid components of TCM with antitumor effects and elaborate their mechanisms of action. Moreover, some Chinese medicines have a high degree of toxicity, and their safety in clinical applications has been called into question, thus limiting the promotion of clinical applications of these Chinese medicines in the international community.

Although a large proportion of Chinese medicines and compound prescriptions still have unclear antitumor mechanisms or toxic reactions (101), most of the Chinese medicines currently used clinically are those with proven safety. Nevertheless, if further developments are to be pursued, the definitive toxicity of herbal medicines has yet to be thoroughly researched (102).

More fundamental studies are necessary to confirm the pharmacological mechanisms of action of Chinese medicines and compounded formulations to achieve wider clinical application and a definite improvement in the quality of life of patients.

Chinese herbal components have the disadvantages of poor absorption, high toxicity, and lack of targeting, which may be complemented by nanomaterials in various aspects to develop safer, more effective, and stable clinical applications. The combination of Chinese medicine and materials science, especially for the integration of the active components of Chinese medicine and nanoparticles, will perhaps serve as a novel breakthrough.

## Author contributions

KS searched for the literature and wrote the manuscript; LW and SW searched for the literature, WD extensively edited the manuscript. All authors contributed to the article and approved the submitted version.

## Funding

This work was supported by the Health System Innovation Project of Shanghai Putuo Science and Technology Commission (No.ptkwws202002), Traditional Chinese Medicine Clinical Key Specialty Construction Project of Shanghai Putuo District (No.ptzyzk2101), Clinical Specialized Discipline of Health System of Putuo District in Shanghai (No. 2021tszk01) and Construction of Colorectal Cancer Special Disease Alliance with Integrated Traditional Chinese and Western Medicine (No. ZY(2021-2023)-0302).

## Conflict of interest

The authors declare that the research was conducted in the absence of any commercial or financial relationships that could be construed as a potential conflict of interest.

## Publisher’s note

All claims expressed in this article are solely those of the authors and do not necessarily represent those of their affiliated organizations, or those of the publisher, the editors and the reviewers. Any product that may be evaluated in this article, or claim that may be made by its manufacturer, is not guaranteed or endorsed by the publisher.

## Footnotes

1 For abbreviations and terminology refer to **Table 1**.
